# Navigating medical school with autism: a systematic review exploring student experiences & support provision in the United Kingdom

**DOI:** 10.1186/s12909-025-06866-9

**Published:** 2025-07-01

**Authors:** Debbie Aitken, Grace Hodge, Michael Page, Ella Lastmann, Sebastian Hiller, Riya E. George

**Affiliations:** 1 Department of Education, University of Oxford, Oxford, OX2 6PY UK; 2 Institute for Academic Development (IAD) Associate, University of Edinburgh, Edinburgh, UK; 3 Faculty of Medicine and Dentistry, Queen Mary University of London, London, UK; 4 MBA Candidate at School of Management, University College London, London, UK; 5 Faculty of Education, University of Cambridge, Cambridge, UK; 6 Faculty of Medicine and Dentistry, Queen Mary University of London, London, UK; 7 University of Edinburgh (Edinburgh Medical School), Edinburgh, UK

**Keywords:** Autism, Medical student, Undergraduate medicine, Student support, Diversity and inclusion, Additional needs, Pastoral care, Neurodivergent, Neurodiversity

## Abstract

**Background:**

Medical education is inherently demanding, requiring students to navigate academically challenging courses, high-stake assessments, clinical training and interpersonal interactions within high stress environments. Autistic medical students can experience profound differences academically and professionally if their support needs are not identified and addressed by specific pedagogic adaptations. This systematic review aims to explore the experiences and support provision for autistic undergraduate medical students in the United Kingdom, through a critical interpretive review of the literature.

**Method:**

The search strategy involved the use of 6 electronic databases, supplemented by citation tracking, consultation with academic experts and library searches. Of 400 papers, 6 satisfied the inclusion and exclusion criteria. Critical interpretive synthesis (CIS) was used to analyse these papers. The study design assimilated methods adopted in conventional systematic reviews within the format of CIS, to combine the entire body of literature and generate theoretical categories.

**Results:**

Three synthetic constructs (overarching themes) were produced from the analysis; ‘reassessing prevailing narratives on autism’, ‘navigation differences’ and the ‘absence of meaningful support provision’. Together these constructs generated a theoretical framework (‘synthesising argument’) defined as ‘reframing support for success’ for autistic and neurodiverse medical students, which collectively explained the findings of the review. ‘Reframing support for success’ provided an explanation for the inconsistencies in understanding the lived experiences of autistic medical students and the required adjustments and support provision. It illustrated the distinct challenges experienced by autistic students in navigating medical school as well the necessity to acknowledge the unique strengths autistic medical students bring.

**Conclusion:**

The review calls for enhanced awareness and training within medical schools to foster an inclusive environment that accommodates neurodiversity more broadly. It advocates for legislative compliance and change in offering reasonable adjustments and stresses the potential benefits of viewing autistic traits as strengths in medical practice.

**Supplementary Information:**

The online version contains supplementary material available at 10.1186/s12909-025-06866-9.

## Introduction

The term "neurodiversity" is frequently misinterpreted or incorrectly used as a substitute for conditions such as autism, dyslexia, or other neurocognitive differences. In reality, neurodiversity encompasses the vast and natural spectrum of cognitive functioning that exists among all individuals. The term "neurodiverse" applies to society as a whole, reflecting this variation across the population. Meanwhile, "neurodivergent" refers to individuals whose cognitive functioning differs from socially established norms—contrasting with the majority, who are considered “neurotypical” [1]. Medical educational institutions have historically been designed for neurotypical individuals [2]. Despite many institutions having overtly inclusive policies, neurodiverse students often find themselves having to navigate a system predominately designed by, and for, neurotypical people.

### Defining autism

Autism is one condition that is included under the umbrella term of neurodiversity. The conceptualisation of autism has evolved significantly from its earlier descriptions and whilst is it still classified as a neurological and developmental disorder, recent evidence and societal movement towards a neurodiversity affirmative approach has occurred. This approach reframes our deficit-based view of autism and other neurodiverse conditions away from that of a disorder towards one of difference and diversity. The National Health Service (NHS) recognises that autism is a spectrum, non-linear in its experience and presentation. Autistic individual differences can range from not having an intuitive concept of social hierarchies, experiencing sensory sensitivities and overload, differences with executive functioning and approaches to social interaction; to a heighted level of creativity, increased attention to detail, exceptional memory and problem-solving skills and having a strong sense of social justice. Little is known about the exact number of autistic medical students in the UK, although available disability data in higher education more broadly shows the number of autistic students has doubled since 2015 to 2020 [3].

However, autism encompasses a broad spectrum of support needs, with significant variability in cognitive, sensory, and communication abilities [4]. While some autistic individuals require minimal support, others, often described as having "profound autism," experience considerable challenges, including limited or no verbal speech and high levels of dependency on caregivers [5]. Estimates suggest that approximately 30–50% of autistic individuals fall into this category, necessitating lifelong assistance in various aspects of daily living [6]. Although those with profound autism are unlikely to enter medical education, their experiences remain an integral part of the autism narrative and should not be overlooked in research and policy discussions. Ensuring that autism advocacy and educational frameworks reflect the full spectrum of autistic experiences helps create more comprehensive and inclusive strategies for supporting neurodivergent individuals in society [7].

### Navigating medical school with autism

Medical educational institutions are increasingly being driven by performance and efficiency. High productivity is expected in compressed time frames and growing demands are placed on medical students year on year. Medical education settings are fraught with high expectations and being ‘overworked’ is now normalised [8]. The disclosure of any form of disability has the potential to raise concerns about a medical student’s professional competence to achieve expected results [9]. Recent reports illustrate that medical education and medical contexts may be regarded as unwelcoming to disabled students and members of faculty, with reports of negative attitudes from peers and faculty, inaccessible work environments, struggling to receive reasonable adjustments and feeling excluded [1, 3]. Whilst there is considerable research on autism within the broader educational context, the experiences of autistic medical students are relatively unexplored and not fully understood.

### Establishing effective support provision

The stressors present in medical educational institutions in meeting the needs of diverse students, and the pressure to do more with less, further complicates and compounds the adverse experiences for autistic medical students [10]. Seeking reasonable adjustments and accommodations is a cumbersome process for students with additional needs, with ambiguous practices, unnecessary bureaucracy and information not readily available being commonly noted [11]. Recent reports showed students with disabilities such as autism also experienced a heavy administrative burden created by applying for, being assess for and organising and chasing up the support they required and needed [12]. The Disability Confident was introduced in the United Kingdom in 2013 [13], with the aim of encouraging employers and employment bodies to be proactive about supporting people with disabilities and neurodiversity. However, there is no evidence to date that this scheme has received a high profile in the university sector and little to suggest that necessary practical changes in the university environment have been maintained to support students with disabilities and neurodiverse students.

### Time for change and action

Medical educational institutions in the UK are recognising that these issues cannot remain unaddressed and the growing interest in supporting students with autism and other neurodiverse conditions is in part stimulated by legislation. Higher educational institutions such as medicine education have a public duty to proactively consider the needs of all students and make reasonable adjustments to ensure equal and meaningful participation. Understanding the unique barriers that some autistic medical students face and the nature and extent of these barriers will assist medical schools in knowing how best to support these students.

A literature review was therefore conducted to explore these issues in greater depth. Methodologies used in systematic literature reviews were utilised to ensure clarity, transparency, and depth in systematically searching, selecting, and synthesising existing knowledge [14]. The aims of this systematic review are to:

- Identify how autism is conceptualised and understood in undergraduate medical education.
- Develop insight into the lived experiences of autistic undergraduate medical students.
- Identify and review the current support provision for autistic medical students and its perceived efficacy.

## Methodology

Although systematic reviews are a well-established method for identifying, appraising and summarising a body of evidence, they are best used where there is a basic homogenous phenomenon and the comparability between studies is sufficient to allow the data to be aggregated for analysis [15]. Definitions of autism are not consistently specified nor operationalised across the medical education. The literature is diverse and complex, using a wide range of research methodologies to explore and discuss this topic. In addition, there are substantial adjunct literatures that contribute to the field and understanding of autism including neurodiversity and disability.

### Critical interpretive synthesis

Critical interpretive synthesis (CIS) is a reflective, flexible methodology, involving the thoughtful interpretation of a broad range of literature, allowing it to be usefully applied to specific and relevant real-life contexts [16–18]. Unlike meta-analytical approaches, CIS goes beyond simple synthesis, allowing the investigation of how a phenomenon and its underlying assumptions are constructed. CIS was particularly appropriate for our study, as the search strategy yielded a wide range of diverse information sources which were relevant to our research aims. Consequently, CIS allowed us to integrate and critically synthesise multiple information sources in an empirically and theoretically grounded way [16].

### Study design

Our study design incorporated conventional systematic review methodology within this CIS framework. Detailed, pre-tested search strategies were used to guarantee replicability, along with clearly defined eligibility criteria to maintain inclusive standards and capture diverse sources of evidence [15]. The study used transparent data extraction processes, a co-rated approach to study inclusion, and a quality appraisal tool to analyse the studies yielded from our search. These three elements sought to minimise personal biases and enhance comparability between studies.

**Fig. 1:**
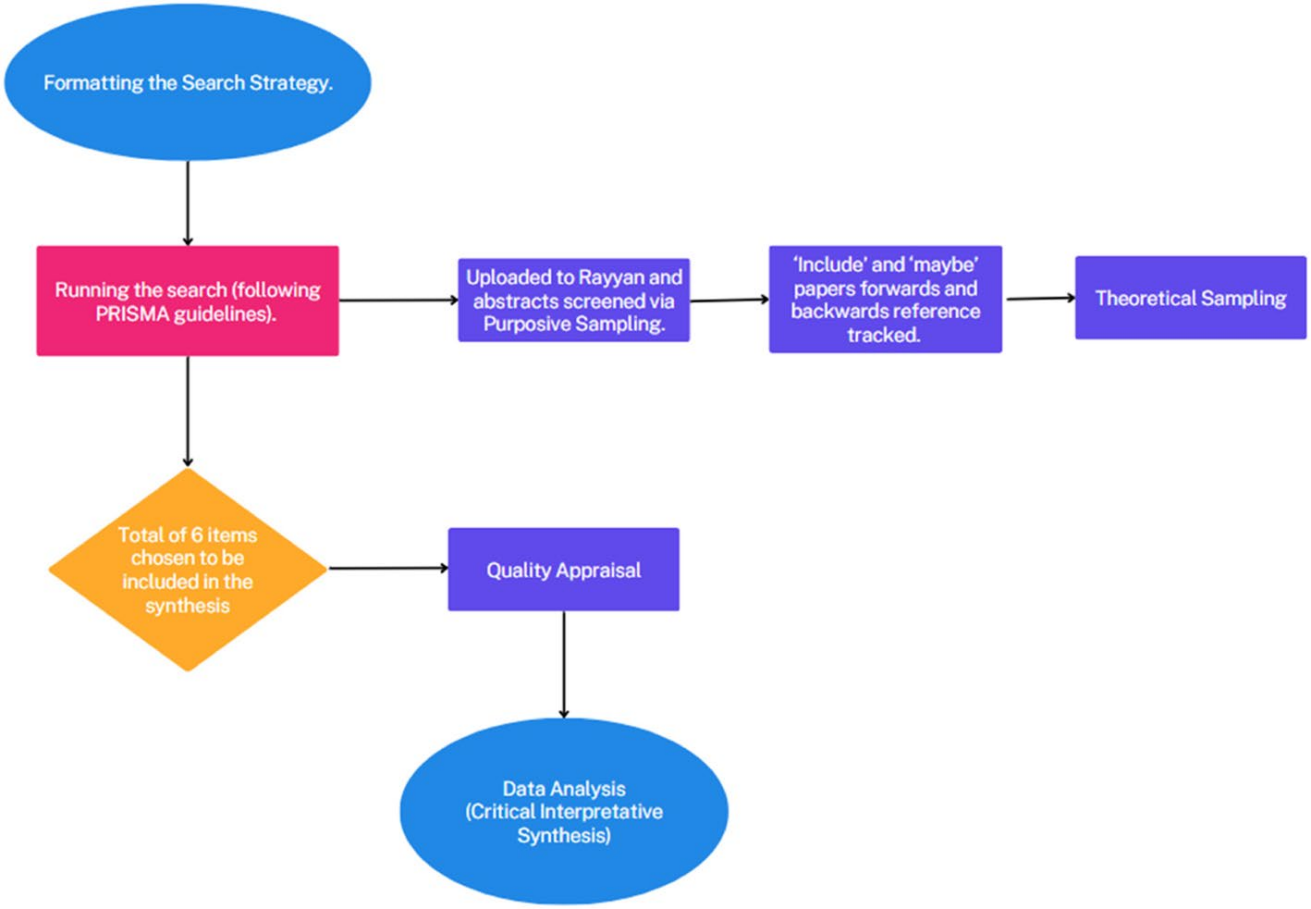
Summary of the study stages

Throughout each stage of our study (formulating the research questions, conducting literature searches, selecting studies, appraising quality, and extracting data) we employed the flexible, iterative approach characteristic of CIS [16]. This integration is crucial for ensuring methodological transparency and improving the accuracy of our conclusions. In the interests of transparency and replicability, each stage of the study is detailed below and supported by Fig. 1.

### Search strategy

Six electronic databases were searched: Scopus, Web of Science, ERIC (via Proquest), MEDLINE, EMBASE and APA PsycINFO.

Our search strategy included free-text and MESH terms, as well as a variety of controlled-vocabulary terms. These terms were variable to account for differences in the indexing of particular databases. To maximise the retrieval of relevant studies, this approach also recognised the existence of multiple related terms and meanings for the words autism, ASD, neurodiversity and disability.

We developed, tested, and adapted pilot searches using synonymous indexing terms tailored to specific databases. The search strategy was developed systematically and refined iteratively through multiple stages. Initially, broad search terms related to autism and medical education were identified and tested. Based on preliminary results, these terms were adjusted to improve specificity and relevance. The process included consultations with information specialists and repeated testing across multiple databases to ensure comprehensive coverage.

Our search strategy was significantly enhanced through reference chaining, consultation with various librarian experts, and by incorporating key terms identified in pertinent studies to boost the empirical applicability. Reference chaining for all relevant items was considered essential, as the limited body of evidence in this field is generally poorly defined and dispersed across various research areas.

The full search on each database was conducted on the 19th May 2024, covering the period from 2010 to June 2024, using the following search terms:

“autis*”, “Autistic Spectrum Disorder”, “ASD”, “Asperger*”, “neurodiver*”, “medical education”, “medic*”, “medical school”, “doctor*”, “medical student*”, “undergraduate medical education”, “staff faculty development training”, “continuing professional development”, “CPD”, “faculty”, “student support”, “university” and “clinical competence”.

The search terms used varied slightly between the different databases to maximise the number of relevant results, as confirmed by several rounds of piloting with librarian support. Complete details on how the search strategy was adapted for each database can be found in Appendix 1. Databases were searched in the title, abstract, and keyword fields, and in the MeSH fields when appropriate MeSH terms existed. Grey literature including trial registries, unpublished studies, relevant websites and thesis repositories were also searched but found nothing of relevance.

### Eligibility criteria

Inclusion criteria aimed to achieve ‘maximum explanatory value’ rather than simply an aggregation of similar concepts [16]. Consequently, preference was given to both conceptual and methodological rigour; meaning preference was given to studies that demonstrated clear theoretical frameworks, robust qualitative or quantitative methodologies, and transparency in data collection and analysis. Eligible studies needed to conform to our inclusion and exclusion criteria, include detailed descriptions of its research design, and a clear articulation of findings that contributed to the understanding of autistic medical students' experiences. Studies lacking methodological clarity or with significant risk of bias were excluded to ensure the reliability and applicability of the findings.

The inclusion and exclusion criteria are outlined below:

Inclusion criteria:

- Scholarly articles and peer-reviewed papers, including both empirical research studies and perspective or opinion pieces, within a time frame from 2010 to June 2024 were included in this review.
- Papers must be in the English language but can be from international sources.
- Peer reviewed papers exploring how autistic medical students are supported in undergraduate medical education in the United Kingdom.

Exclusion criteria.

- Scholarly articles and peer reviewed papers not written in English or those published before 2010 and outside the UK were excluded.
- Peer review or scholarly articles that explored autism in school children, non-medical university students, or non-medical health professionals (e.g. nurses, physiotherapists) were excluded.

### Study selection

Three independent reviewers, with expertise in medical education and neurodiversity research, conducted the study selection process. Reviewers were selected based on their familiarity with systematic review methodologies and underwent training on the inclusion and exclusion criteria to ensure uniformity in decision-making. To assess interrater agreement, an initial calibration exercise was conducted, where a subset of studies was reviewed independently and compared for consistency. Regular consensus meetings were held to resolve discrepancies and refine the selection process.

**Fig. 2:**
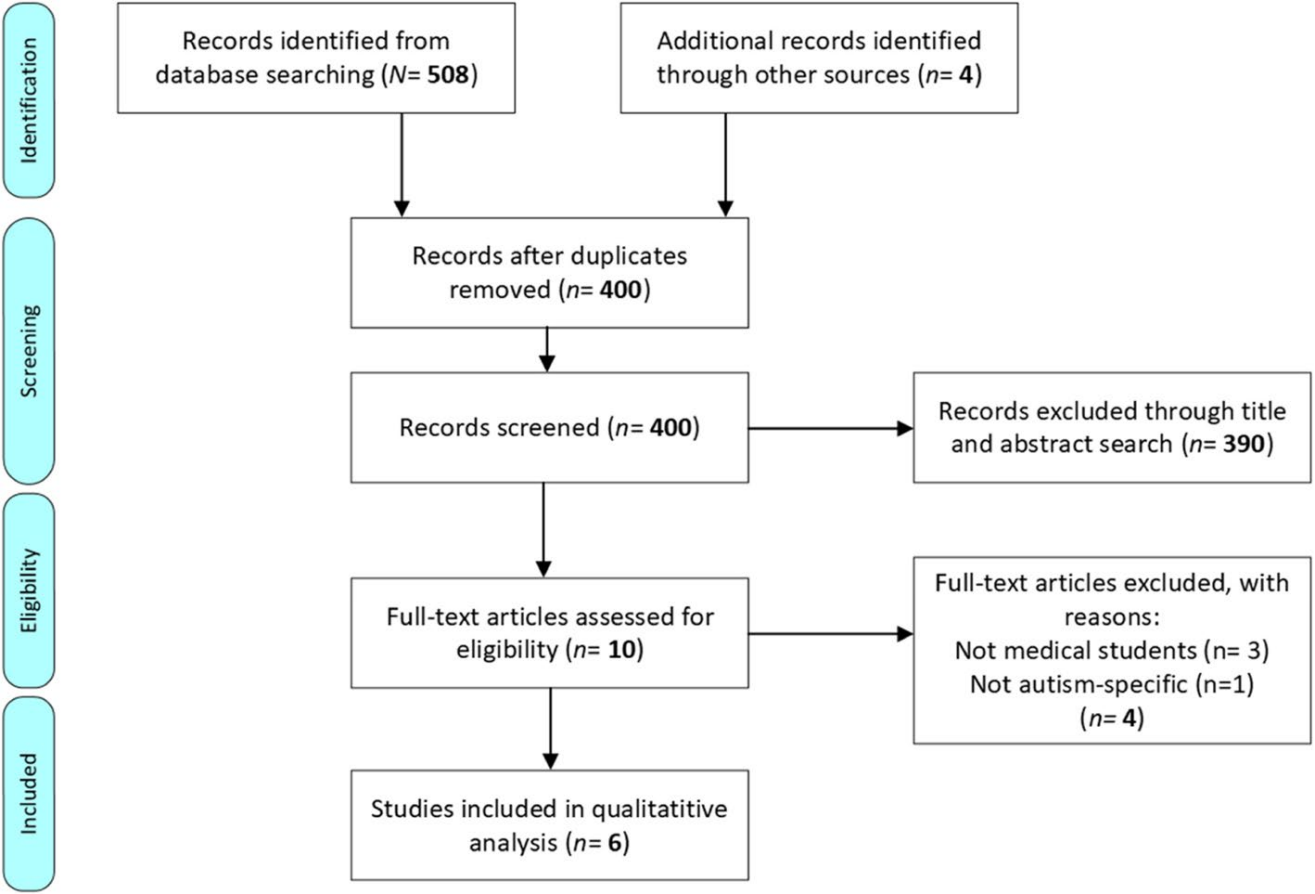
PRISMA (Preferred Reporting Items for Systematic reviews and Meta-Analyses) flowchart of the literature search

Search results were uploaded to the screening application Rayyan and the abstract of each paper was screened. Studies were then apportioned to an “include”, “maybe” and “exclude” category. The bibliographies of each paper in the “include” and “maybe” groups were forwards and backwards reference tracked. Full text PDFs were then retrieved and evaluated based on their quality. The search strategy and study selection were designed, performed and outlined using the Preferred Reporting Items for Systematic Reviews and Meta-analyses (PRISMA) guidelines (Fig. 2) below.

**Fig. 3:**
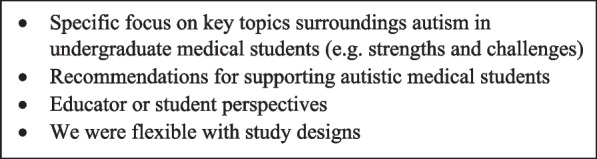
Study Selection Criteria

A total of 400 unique items were found. We employed purposive sampling in accordance with the established inclusion and exclusion criteria to focus on the retrieval of items that explicitly detailed the experiences of autistic undergraduate medical students in the UK within in the title or abstract. Following this, theoretical sampling was utilised, selecting items based on their potential to address the review question, to advance the emerging analysis (Fig. 3) [17]. This iterative process of selecting items continued throughout the analytical stage. A total of 6 items, from a time span from 2010—2024, were chosen to be included in the synthesis.

### Quality appraisal

CIS does not formally evaluate the quality of literature, as a single quality appraisal tool would be inadequate for assessing diverse items and heterogeneous study designs. To address this limitation of traditional CIS, in this study, we used a structured quality appraisal checklist developed by Hawker et al. [19]. This checklist provides a transparent scoring system for each item, akin to the appraisal of randomised control trials, and has proven beneficial for CIS by prompting reviewers to clearly articulate the rationale of their judgments [19, 20]. Three independent reviewers collaborated to determine the eligibility and quality of each study, however weaker methodological papers that could offer theoretical insights were included [21].

### Data analysis

CIS involves a data analysis process that resembles qualitative research analytical processes and seeks to generate a theoretical output in the form of a synthesising argument. This argument serves as a coherent theoretical framework that encapsulates the overall findings. We adopted the standard method for data extraction analysis as described by Dixon-Woods et al. [16]. Key information from each item was extracted and formulated, as illustrated in Table 1.

**Table 1:** Characteristics of 6 Studies Included

Study	Type of Paper	Methodology	Themes	How was this paper found?
Shaw et al. [21]	Scholarly perspective/letter to the editor	Correspondence/ letter to the editor (The Lancet)	• Strengths of autistic doctors (viewed as an asset not a hindrance) • Recommend better awareness and training of staff	Scopus
Magnin et al. [22]	Research Article	Anonymous cross-sectional electronic survey. Quantitative descriptive statistics and qualitative analysis of free text	• Relative lack of information about NDDs (term used by authors), but autism as most problematic and as a hinderance in teaching/education, autistic medics as risk to future patients, medical teachers need specific training about pedagogic management and advice • Medical teachers would be interested in specific training and procedures about the pedagogic ‘management’ of NDDs students • Diagnosis framed as a 'double-edged sword'—beneficial adjustments versus 'social burden'	Reference tracked from Shaw et al. [23]
Moore et al. [24]	Lived experience commentary	Correspondence/ letter to the editor (The Lancet)/ Lived experience commentary	• Strengths of autistic doctors • Need for greater awareness, understanding and support, • Focus on student-led reasonable adjustments (not just generic ones for all)	Reference tracked Magnin et al. [22] to find Price et al. [25]. Searched Price et al. [25] in Scopus and clicked ‘cited by’ to find this paper
Giroux & Pélissier-Simard [26]	Review	Research Review	• Autistic traits: strengths and challenges for learning, teaching and clinical practice • Value of a ‘neurodiversity lens’ for educators • Awareness and understanding of the how autistic traits may influence the challenges faced by struggling medical students • Contributors to the ‘blind-spot’ of understanding autistic traits in medical students • The increased importance of considering autistic traits in medical education	Reference tracked from Shaw et al. [27]
Shaw et al. [27]	Scholarly perspective	Perspective piece/ correspondence/ letter to the editor	• Strengths of autism • Perspective of a large group of autistic doctors (ADI), explaining the need to do away with the tragedy narrative and encourage the required variety of teaching/educational methods and environment to be more accessible and inclusive • The role of positive role modelling is very important, as well as overall increased awareness which is currently deficient	Reference tracked from Shaw et al. [23]
Shaw et al. [28]	Research Article	Interpretive phenomenological study	• Autism stereotypes, stigma and discrimination • Masking (the need and impact) • Social isolation and bullying but the importance of autistic role models • Navigating the system and dilemmas of diagnosis disclosure • Need for (and lack of) reasonable adjustments • Strengths and challenges	Scopus

## Results

**Fig. 4:**
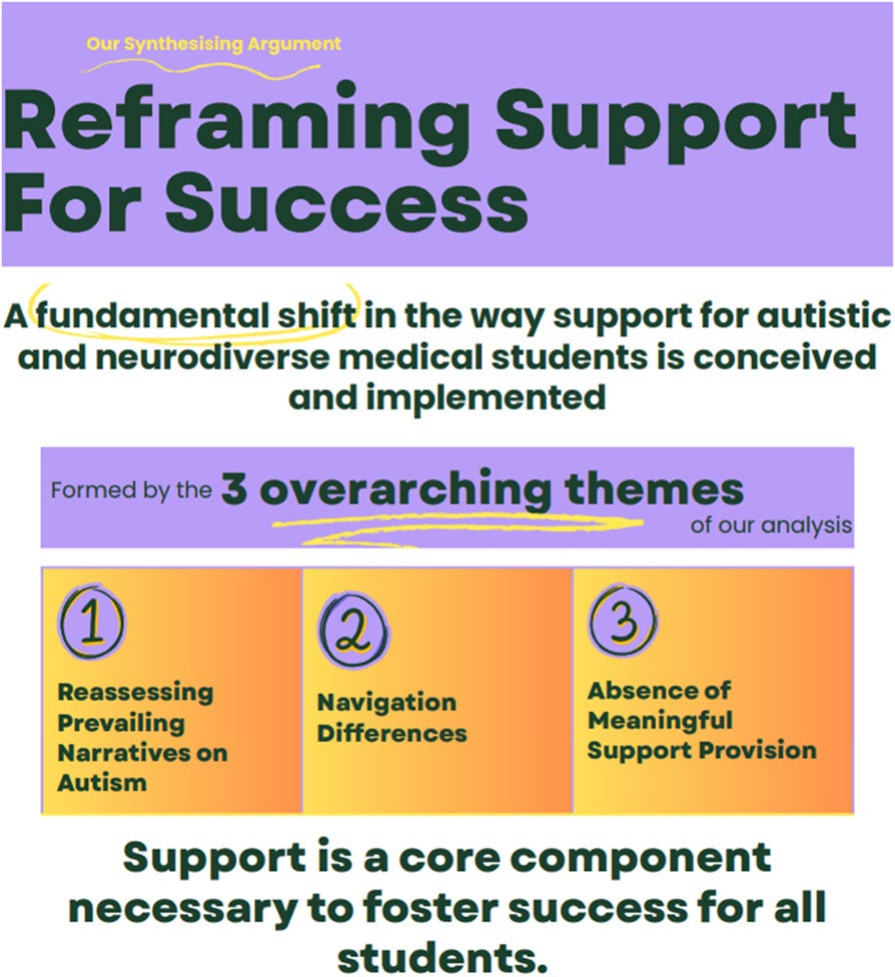
Reframing Support for Success

Three synthetic constructs (overarching themes) were produced from the analysis; ‘reassessing prevailing narratives on autism’, ‘navigation differences’ and the ‘absence of meaningful support provision’. Figure 4 presents the theoretical framework (‘synthesising argument’) generated by these overarching themes, defined as ‘Reframing Support for Success’, which emerged from the analysis of support for autistic and neurodiverse medical students. This framework is underpinned by the three overarching themes: 1) reassessing prevailing narratives on autism, 2) navigation differences, and 3) the absence of meaningful support provision. These themes collectively explain the key findings of the review and highlight the need for a fundamental shift in how support is conceived and implemented for autistic medical students. Figure 4 visually represents these constructs, illustrating their role in shaping a reframed approach to support.

### Reassessing prevailing narratives on autism

One of the key themes across several papers is that autistic medical students often choose not to disclose their diagnosis due to fears of stigma and discrimination. This key theme illustrates the complex dynamics surrounding autistic medical students, especially in terms of disclosure, identity, and systemic biases. Shaw et al. [28] reported that these students navigate a complex tension between their self-identity and external perceptions, frequently confronting stereotypes and stigma. The study highlighted both their strengths, such as a strong commitment to helping autistic patients, and their challenges, like uncertainty and difficulties in social interactions. Stereotypes persisted among faculty, including those on fitness-to-practice panels, leading to discriminatory and punitive actions, such as a lack of understanding and exclusion from programs. Shaw et al. [28] emphasize the harmful nature of these stereotypes, while Magnin et al. [22] suggest that autism is viewed as a psychiatric condition, likening it to schizophrenia. They argue that medical teachers see autistic students as potential risks to patients, framing the need to adapt medical education as an ethical dilemma within a system focused on patient care. Further, Moore et al. [24] discussed the central tension autistic students face when considering whether to seek a diagnosis: the potential for stigma and discrimination versus the chance to receive practical and emotional support. On the one hand, a diagnosis could open doors to support systems that help with both their personal and academic struggles. On the other hand, disclosing this diagnosis might expose them to increased discrimination, further perpetuating the stigmatization of autism within medical education systems.

This theme underscores the need for more inclusive educational environments where autistic students can thrive without fear of stigma, and where their unique strengths are acknowledged and supported.

### Navigation differences

Autistic medical students often face unique navigational challenges in their educational environments, as highlighted in studies like Shaw et al. [28]. These challenges stem from sensory sensitivities, difficulties in social interactions, and the constant pressure to conform to non-autistic norms. Shaw et al. [28] reported that sensory issues significantly impacted the learning experiences of autistic students. Participants described how environmental factors, such as noise in lecture theatres or clinical settings, were often overwhelming and physically painful, hindering their ability to focus and learn effectively. Despite this, reasonable adjustments were frequently lacking, and students were sometimes advised or pressured to take time out instead. To fit in with non-autistic peers, many autistic students engaged in masking, which involved suppressing their autistic traits to avoid stigma. This was exhausting and led to considerable mental and emotional strain. Moore et al. [24] also highlighted the burdens of masking on autistic individuals, while Magnin et al. [22] focused more on its impact on others, including students, teachers, and patients.

Shaw et al. [28] also found that autistic students often felt socially isolated and struggled with social interactions with peers and staff. This isolation was worsened by bullying and a lack of understanding from others, further alienating them from their educational environment. Connecting with autistic peers and faculty role models provided some relief. Students felt pressured to mask their autistic traits to conform to an environment with little to no adjustments, as "non-autistic" behaviour was often equated with professionalism. Shaw et al. [28] argued that this expectation represented a hidden curriculum, which excluded autistic students from the accepted norms of a medical student identity.

Systemically, autistic students felt that disclosing their diagnosis in medical school was risky. Many felt misled by the lack of support they received after disclosing their status, despite being assured otherwise. Resilience was often "weaponized" against them, and reasonable adjustments were either not made or were generic and unsuitable, even when medically necessary. Moore et al. [24] called for increased awareness, understanding, and support, emphasizing the need for student-led, rather than generic, reasonable adjustments. They also stressed that such adjustments are crucial for allowing autistic doctors to have successful careers.

The implications of these findings are significant, both for individual autistic students and for medical institutions. The lack of suitable accommodations for sensory sensitivities and the expectations around masking point to systemic shortcomings in inclusivity. Autistic students are being placed in environments that not only fail to support their needs but also actively contribute to their stress and isolation. The studies call for a shift in the way medical education is structured, emphasizing the importance of tailored adjustments that address the specific needs of autistic students.

By addressing these navigational differences, medical education can become more inclusive, creating an environment where autistic students can thrive without the need to mask their identities or endure sensory overwhelm. This would not only benefit the students themselves but also enrich the profession, allowing a more diverse range of doctors to contribute their unique perspectives and strengths to patient care.

### Absence of meaningful support provision

The lack of meaningful support for autistic medical students is a significant theme in recent research, with studies like Magnin et al. [22] highlighting the limitations in current medical education systems. One of the central issues raised is the insufficient knowledge among medical educators about neurodevelopmental disorders, particularly autism. This knowledge gap prevents educators from providing effective support, creating an environment where autistic students are often seen as "risks" rather than capable contributors.

Magnin et al. [22] discussed the broader issue of limited information about neurodevelopmental disorders in medical education, emphasizing that the lack of knowledge about autism is particularly problematic and hinders education. They portrayed autistic medical students as potential risks to future patients, recommending that medical teachers receive specific training on pedagogic management. Their findings indicated that medical educators believe many autistic students will struggle in their careers and feel unprepared to support them, leading to a strong interest in specialized training and procedures.

The study by Magnin et al. [22] is framed within a medical model, using language like "management" of students, akin to managing patients. They suggest that without appropriate adaptations, autistic students are "at risk," proposing that these students should receive accurate diagnostic information to guide them toward an "adapted career pathway," which could imply steering them away from clinical medicine. This approach not only diminishes the potential of autistic individuals but also reinforces the notion that autism is a barrier to success in medicine, rather than a different way of processing information and engaging with the world. This perception of autistic students as "imperfect" is in stark contrast to the idealized image of the "perfect mind" in medicine, described as intelligent, empathetic, hard-working, and psychologically stable. Such dichotomous thinking frames autism as a deviation from this ideal and reinforces the stigmatization of autistic individuals within medical education.

In contrast, Shaw et al. [28, 21] advocate for greater understanding and support from medical schools, urging that autistic doctors be viewed as assets rather than hindrances. They call for the inclusion of neurodiversity-affirmative autism training in medical curricula and argue that autistic medical students have valuable contributions to offer, such as a strong sense of ethics, professionalism, and a non-judgmental perspective on difference. Shaw et al. [28] also see recent General Medical Council (GMC) – the public body that maintains the official register of medical practitioners within the United Kingdom—policies as opportunities for improvement, emphasizing the need for active inclusion of autistic students in medical education. Although some assertions in their paper lack a strong evidence base, subsequent empirical studies by Shaw and colleagues recommend using an autism-specific framework (the ‘SPACE’ framework) to guide reasonable adjustments for autistic individuals in healthcare settings. Additionally, Shaw et al. [27] highlight the importance of positive role modelling, advocating for diverse teaching methods and environments to create a more accessible and inclusive medical education.

The absence of meaningful support and accommodations has profound implications for autistic medical students. Without proper adjustments, these students are placed in environments where they are *set up to struggle rather than thrive*. The framing of autism as a liability instead of an asset reinforces exclusionary practices that hinder the careers of autistic individuals in medicine. The lack of support also leads to high levels of emotional strain as these students must navigate an educational system that does not recognize their unique needs.

Implementing targeted support measures, such as specialized autism training for medical educators, the adoption of neurodiversity frameworks, and the use of diverse teaching methods, can foster a more inclusive environment. These measures would not only support the academic and professional success of autistic students but also challenge the discriminatory perceptions that currently exist in medical education. Recognizing the contributions that autistic doctors can make to the profession—such as offering different perspectives on patient care and being role models for autistic patients—can help shift the focus from "managing" autistic students to supporting and empowering them.

The shift toward neurodiversity-affirmative training and policies will ultimately lead to a more inclusive medical field, where differences are not only accepted but valued. This would help autistic students develop their full potential in their medical careers, benefitting both them and the broader healthcare system.

### Reframing support for success

The overarching synthetic argument is termed reframing support for success this emphasizes a fundamental shift in the way support for autistic medical students is conceived and implemented. Rather than viewing support as an optional or remedial measure—a simple "additive" to the existing system—it is reframed as a core component necessary to foster the success of all students, including those with autism. This reframing highlights the importance of creating an inclusive and adaptable learning environment that acknowledges the diverse strengths and needs of autistic students.

**Fig. 5:**
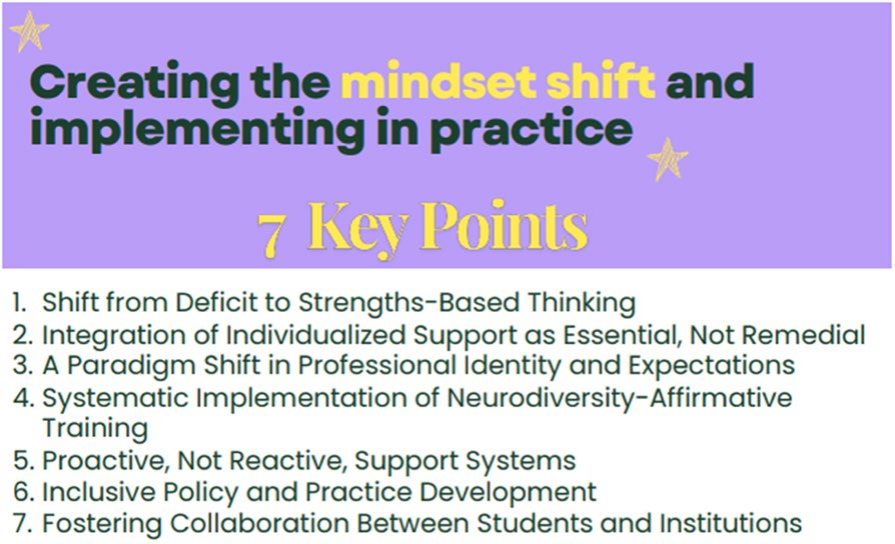
Key Points for mindset shift and implementation

Figure 5 outlines the practical steps required to implement this reframing, presenting seven key points that facilitate a shift from deficit-based approaches to strengths-based, neurodiversity-affirmative support. These points emphasise the need for systemic changes, including proactive support systems, inclusive policy development, and fostering collaboration between students and institutions.

#### 1. Shift from Deficit to Strengths-Based Thinking

The prevailing mindset in many medical institutions sees support for autistic students through a deficit lens, where these students are viewed as needing extra help because they fall short of an ideal. To reframe support for success, there needs to be a shift toward strengths-based thinking. Autistic students often bring unique abilities, such as heightened attention to detail, strong ethics, and deep empathy, especially toward patients with neurodevelopmental disorders. Instead of focusing solely on their challenges, institutions should recognize and build upon these strengths to promote success, both academically and professionally.

In practice:

- Neurodiversity should be integrated as a core component of medical training, ensuring that autistic traits are better understood within the profession. While some autistic traits may bring valuable strengths to medical practice, others may require appropriate support and reasonable adjustments from the system and colleagues to facilitate success. A balanced approach that acknowledges both the potential contributions and the challenges autistic individuals may face is essential for fostering an inclusive medical education environment.
- Implement training programs that help educators recognize and nurture the strengths of autistic students.

#### 2. Integration of Individualized Support as Essential, Not Remedial

Support for autistic students should not be viewed as something “extra” or a remedial accommodation for those who are struggling. Instead, individualized support must be seen as essential to fostering an environment where all students can succeed, similar to how different learning styles are catered to. This can include personalized learning plans, sensory adjustments, flexible assessment methods, and mentoring programs tailored to each student's needs.

In practice:

- Schools should adopt frameworks like the "SPACE" framework [29], which provides structured, autism-specific guidance on how to improve healthcare experiences for autistic individuals by addressing five key areas: sensory needs, predictability, acceptance, communication, and empathy. Its goal is to reduce healthcare disparities and enhance outcomes by creating a more supportive and inclusive environment for autistic patients, but this could be adapted for medical education environments.
- Institutions should promote a culture where all students are offered individualized support as part of their educational journey, reducing the stigma around needing accommodations.

#### 3. A Paradigm Shift in Professional Identity and Expectations

Many autistic students are pressured to conform to an idealized version of a medical professional, which often excludes neurodivergent behaviours and traits. This creates an invisible barrier, a hidden curriculum, where masking and suppressing autistic traits are seen as necessary for professionalism. To reframe support for success, the medical community needs to redefine what it means to be a successful medical professional, embracing neurodiversity as part of professionalism.

In practice:

- Institutional policies should explicitly acknowledge that professionalism is not inherently synonymous with neurotypical behaviors. While certain autistic traits can be assets in medical practice, others may necessitate reasonable adjustments or structured support. Professionalism in medicine involves a range of learned behaviors, expectations, and adaptive strategies that all medical professionals, regardless of neurotype, must develop. Ensuring that professional standards are inclusive and flexible can help foster an environment where autistic medical students can thrive without unnecessary barriers.
- Encourage role modelling and mentorship from autistic or neurodivergent doctors, demonstrating that successful medical professionals come in many forms.

#### 4. Systematic Implementation of Neurodiversity-Affirmative Training

To create this shift, educators and staff must be trained to understand and embrace neurodiversity, moving away from the medical model of "managing" autistic students and toward a neurodiversity-affirmative approach. This involves training medical educators on the strengths and challenges of autistic students, understanding sensory and communication needs, and promoting inclusive teaching practices. Neurodiverse educators and staff should also have access to the same training opportunities to enhance their ability to support students and colleagues effectively. Ensuring that all individuals, regardless of neurotype, are equipped with the knowledge and skills to promote inclusion will contribute to a more supportive and equitable medical education environment.

In practice:

- Mandatory neurodiversity training for all faculty, not just for those who directly supervise autistic students. This would ensure that everyone in the academic environment is aware of how to support neurodiverse learners effectively.
- Include neurodiversity content in medical curricula to raise awareness among future healthcare professionals, creating a more inclusive generation of doctors.

#### 5. Proactive, Not Reactive, Support Systems

Support systems for autistic students should be proactively integrated into the structure of medical education, rather than being provided only when issues arise. Often, students are required to disclose their autism diagnosis to receive accommodations, which places the burden on them. Instead, institutions should create inclusive policies that assume diversity as the norm, offering support structures from the outset to all students.

In practice:

- Create learning environments that naturally incorporate sensory adjustments, flexible deadlines, and various assessment methods without requiring formal disclosure of a diagnosis.
- Build mentorship and peer support programs that are open to all students, allowing neurodiverse students to benefit from networks of support without having to advocate for themselves continuously.

#### 6. Inclusive Policy and Practice Development

Shaw et al. [28] emphasized that institutional policies, such as those developed by the GMC, provide opportunities to advance neurodiversity in medical education. Reframing support for success requires policy changes that actively incorporate neurodiversity, ensuring that these policies are not just theoretical but are implemented in day-to-day practice.

In practice:

- Incorporate neurodiversity-focused guidelines into accreditation and evaluation frameworks for medical schools, ensuring compliance with inclusive education standards.
- Develop clear protocols for addressing bullying, isolation, and stigma against autistic students, enforcing consequences for discriminatory behaviours.

#### 7. Fostering Collaboration Between Students and Institutions

Finally, student-led adjustments and feedback mechanisms are essential to ensure that the support systems in place are not only adequate but effective. Institutions should collaborate with autistic students who choose to disclose their diagnosis while also ensuring support mechanisms exist for those who do not disclose. This means actively listening to students' experiences and adjusting policies based on their input.

In practice:

- Regular feedback loops where autistic students can share their experiences with faculty and administrators, ensuring continuous improvement in support structures.
- Empowering students to advocate for themselves and others by creating safe spaces for discussion, while also offering institutional support in these advocacy efforts.

Reframing support for success moves beyond the idea that accommodations are just "add-ons" to help struggling students. Instead, it advocates for a systemic shift toward inclusive environments where neurodiverse students, including those with autism, are empowered to succeed. This requires embracing neurodiversity as a strength, ensuring proactive support, and fostering a medical education system where individualized accommodations are seen as essential to success, not exceptional.

We found evidence of oversimplying or generalising autism discourses across all the papers studied, where autistic individuals were often viewed as inherently capable or incapable of certain tasks solely due to their autism. Negative discourses, rooted in the medical model, were prevalent in some literature, framing autistic students as having a “severe psychiatric condition,” being “at risk,” and requiring “management” through “adaptations.” This approach pathologised and dehumanised autistic students, subjecting them to the educational equivalent of Foucault’s ‘medical gaze’ [30].

This negative discourse is significant because it suggests that fear of stigma and discrimination may deter students from seeking or disclosing an autism diagnosis, preventing them from accessing necessary support and accommodations. Ironically, for those who do disclose, their diagnosis may be used against them as evidence that they are unlikely to succeed in clinical medicine.

In contrast, some papers framed autism positively, highlighting the strengths and abilities of autistic individuals. This perspective challenges the dominant medicalised discourse that perpetuates a deficit model of autism. However, there is a risk of replacing one stereotype with another, where autistic students are either demonised as fundamentally incompatible with safe medical practice or valorised to the extent that the real challenges posed by the educational environment are overlooked. The reality is that autistic people are as diverse in their personalities, capabilities, and preferences as anyone else***,*** reflecting the original intent of the term neurodiversity [31].

## Discussion

Our review makes it clear that autistic medical students often experience significant tensions and contradictions as they navigate undergraduate medical education. These curricula, which are primarily designed with neurotypical assumptions, pose unique challenges for neurodivergent students. Moreover, autistic students frequently discover that they cannot always trust the information they receive about the education system, including elements supposedly designed to support them. This mistrust is exacerbated by evidence suggesting that neurotypical behaviours have been embedded within normative concepts of professionalism, leading to autistic students being perceived as unprofessional when they express their natural ways of being. As a result, many autistic students resort to masking—attempting to present a neurotypical identity—which exacts a significant emotional and psychological toll.

Another factor driving masking is the lack of reasonable adjustments offered to autistic students. The educational environment, especially clinical settings where they work and learn, is often seen as fundamentally unchangeable. This approach, in our view, is inconsistent with current UK legislation. The needs of autistic medical students should be recognised and appropriately addressed in the implementation of the medical curriculum, including during clinical placements.

Building on the observations above, all of the included papers emphasised the need for greater awareness of autism within undergraduate medical education, particularly in university systems and processes like fitness to practise. Most recommended adopting an autism-affirmative approach that views autism as a potential strength. There is a consensus in the literature that autistic medical students are, or could be, valuable assets to medicine and society. However, autism remains poorly understood, and existing training often fails to shift attitudes. Some papers suggested that increased awareness could lead to better outcomes, and given the shortcomings of some faculty development initiatives, the majority of the literature called for more holistic and thoughtful approaches to training.

To synthesise, this review suggests that the current support for seeks to accommodate autistic medical students, at best. We, however, care about how to support autistic medical students for success and flourishing. Viewing autistic students as assets, valued colleagues and team members is an integral step towards achieving this level of genuine support for students’ wellbeing and academic accomplishments. The next step is therefore raising awareness of the strengths of autistic medics and integrating this awareness into the culture of medical schools. A culture shift towards the valuing and active encouragement of autistic medics may have positive snowball effects on the social isolation experienced by many autistic medical students and the perceived expectation of ‘masking’. Improving these aspects of the autistic medical student experience may subsequently improve overall wellbeing and promote curriculum and placement engagement, and thus future success by extension.

To our knowledge, this is the first review to examine the experiences of autistic medical students within the UK undergraduate curriculum. We conducted a comprehensive database search using an iterative process with input from experienced information management professionals and adhered to established guidelines for systematic reviews.

However, our review was limited by the currently small evidence base and the lack of empirical studies in this area. The diversity of the articles also prevented statistical analysis. Despite this, the rigor of our qualitative narrative review was strengthened by multiple rounds of review and discussion among the research team to reach consensus on paper inclusion and the development of codes and themes.

### Bridging Barriers: Considering the Broader Literature

The challenges faced by autistic medical students reflect broader trends in the literature on disability in medical education. Research has consistently highlighted the barriers that disabled students encounter, including inaccessible curricula, inflexible assessments, and a lack of appropriate accommodations [32, 33]. Similar to autistic students, those with other disabilities often find themselves navigating educational environments that prioritize neurotypical and able-bodied norms, reinforcing a hidden curriculum that defines professionalism in exclusionary ways [34]. These systemic barriers contribute to the underrepresentation of disabled students in medicine and may discourage disclosure, as students fear being perceived as incapable of meeting the demands of clinical training [35].

The need for a cultural shift in medical education towards inclusivity is echoed in broader disability advocacy efforts. Studies have emphasized the importance of a social model of disability, which shifts the focus from individual impairments to structural changes that enable full participation [36, 37]. This aligns with calls from the wider disability rights movement, which advocates for reasonable adjustments not merely as accommodations but as essential components of equity in education [38]. Implementing universal design principles—such as flexibility in assessments and adjustments in clinical training—has been shown to benefit all students, not just those with disabilities, by fostering a more inclusive learning environment [39].

By recognizing the shared experiences of autistic students and other disabled medical learners, medical education can move toward a more holistic and affirming framework that values diversity as a strength. Addressing these barriers through faculty training, curriculum redesign, and institutional policy changes will be essential in ensuring that all students, regardless of disability, can succeed and contribute meaningfully to the medical profession.

## Conclusion

The literature clearly shows that autistic students have much to offer medicine, but systemic barriers like lack of understanding, stereotyping, the hidden professionalism curriculum, and inadequate reasonable adjustments hinder their inclusion in the medical community.

To address these issues, faculty development must tackle negative stereotypes and the perception that autistic students are fundamentally incompatible with medical practice and professionalism. An inflexible approach forces students to mask, often at great personal cost. Given the prevailing negative discourse around autism in medical education, we recommend that faculty development programs adopt a neurodiversity-affirmative philosophy, which should also be reflected in university systems and processes, including fitness to practise procedures.

In the UK, autistic students are legally entitled to reasonable adjustments that enable their participation in the curriculum. We recommend increased efforts to remove barriers, especially during clinical placements. Further empirical research into the lived experiences of autistic medical students is urgently needed, given the scarcity of studies in this area. In calling for more research, we stress the importance of moving away from the deficit model of autism, which is prevalent in academic literature, and toward a more balanced and nuanced understanding of autistic individuals’ lived experiences.

## Supplementary Information

Supplementary Material 1.

## Data Availability

No datasets were generated or analysed during the current study.

## References

[1] Shaw SCK, Brown MEL, Jain NR, et al. When I say … neurodiversity paradigm. Med Educ. 2024; 1–3. 10.1111/medu.15565

[2] Olsen J, Griffiths M, Soorenian A, Porter R. Reporting from the Margins: Disabled Academics Reflections on Higher Education. Scand J Disabil Res. 2020;22(1):265–74.

[3] TASA: Transforming Access and Student Outcomes in Higher Education. What works to reduce equality gaps for disabled students [Internet]. University of Lincoln; 2023 Feb. Available from: https://taso.org.uk/wp-content/uploads/TASO-report-what-works-to-reduce-equality-gaps-for-disabled-students.pdf

[4] Lord C, Elsabbagh M, Baird G, Veenstra-VanderWeele J. Autism spectrum disorder. The Lancet. 2018;392(10146):508–20. 10.1016/S0140-6736(18)31129-2 PMC7398158 30078460

[5] Courchesne E, Pramparo T, Gazestani VH, et al. The ASD Living Biology: From Early Development to Neurophysiology, Treatment, and Outcome. Curr Opin Neurol. 2020;33(2):117–32.31743236

[6] Anderson DK, Liang JW, Lord C. Predicting young adult outcome among more and less cognitively able individuals with autism spectrum disorders. J Child Psychol Psychiatry. 2014;55(5):485–94. 24313878 10.1111/jcpp.12178 PMC5819743

[7] Pellicano E, Bölte S, Stahmer A. The hidden inequalities of autism: Why we need to reconsider the ‘spectrum’ concept. Autism. 2018;22(5):483–5.

[8] General Medical Council. The state of medical education and practice in the UK: Workplace experiences. 2024 Aug. Available from: https://www.gmc-uk.org/-/media/documents/somep-workplace-report-2024-full-report_pdf-107930713.pdf

[9] Dolan VLB. ‘…but if you tell anyone, I’ll deny we ever met:’ the experiences of academics with invisible disabilities in the neoliberal university. Int J Qual Stud Educ. 2021;36(4):689–706.

[10] Mellifont D, Smith-Merry J, Dickinson H, Llewellyn G, Clifton S, Ragen J, et al. The ableism elephant in the academy: a study examining academia as informed by Australian scholars with lived experience. Disability & Society. 2019;34(7–8):1180–99.

[11] Stone SD, Crooks VA, Owen M. Going through the back door: Chronically ill academics’ experiences as ‘unexpected workers.’ Soc Theory Health. 2013;11(2):151–74.

[12] Stefani L, Matthew B. The difficulties of defining development: A case study. Int J Acad Dev. 2002;7(1):41–50.

[13] UK Government. Disability Confident campaign [Internet]. London: GOV.UK; [accessed 2025 Feb 03]. Available from: https://www.gov.uk/government/collections/disability-confident-campaign

[14] Arksey H, O’Malley L. Scoping studies: towards a methodological framework. Int J Soc Res Methodol. 2005;8(1):19–32.

[15] Mays N, Pope C, Popay J. Systematically reviewing qualitative and quantitative evidence to inform management and policy-making in the health field. J Health Serv Res Policy. 2005;10(1_Suppl):6–20. 16053580 10.1258/1355819054308576

[16] Dixon-Woods M, Cavers D, Agarwal S, Annandale E, Arthur A, Harvey J, et al. Conducting a critical interpretive synthesis of the literature on access to healthcare by vulnerable groups. BMC Med Res Methodol. 2006;6(1):35. 16872487 10.1186/1471-2288-6-35 PMC1559637

[17] Flemming K. Synthesis of quantitative and qualitative research: an example using Critical Interpretive Synthesis. J Adv Nurs. 2010;66(1):201–17. 20423445 10.1111/j.1365-2648.2009.05173.x

[18] Heaton J, Corden A, Parker G. ‘Continuity of care’: a critical interpretive synthesis of how the concept was elaborated by a national research programme. Int J Integr Care. 2012 Apr 13 [cited 2024 Sep 11];12(2). Available from: 10.5334/ijic.794/10.5334/ijic.794 PMC3429143 22977421

[19] Hawker S, Payne S, Kerr C, Hardey M, Powell J. Appraising the Evidence: Reviewing Disparate Data Systematically. Qual Health Res. 2002;12(9):1284–99. 12448672 10.1177/1049732302238251

[20] Sandelowski M, Barroso J. Finding the Findings in Qualitative Studies. J of Nursing Scholarship. 2002;34(3):213–9. 10.1111/j.1547-5069.2002.00213.x 12237982

[21] Shaw SCK, Doherty M, McCowan S, Davidson IA. Challenging the exclusion of autistic medical students. Lancet Psychiatry. 2022;9(4): e18. 35305752 10.1016/S2215-0366(22)00061-X

[22] Magnin E, Ryff I, Moulin T. Medical teachers’ opinions about students with neurodevelopmental disorders and their management. BMC Med Educ. 2021;21(16):1–9. 33407399 10.1186/s12909-020-02413-w PMC7789168

[23] Shaw SCK, Fossi A, Carravallah LA, Rabenstein K, Ross W, Doherty M. The experiences of autistic doctors: a cross-sectional study. Front Psychiatry. 2023;14:1160994. 10.3389/fpsyt.2023.1160994 PMC10393275 37533891

[24] Moore S, Kinnear M, Freeman L. Autistic doctors: overlooked assets to medicine. Lancet Psychiatry. 2020;7(4):306–7. 32199503 10.1016/S2215-0366(20)30087-0

[25] Price S, Lusznat R, Mann R, Locke R. Doctors with Asperger’s: the impact of a diagnosis. Clin Teach. 2019;16(1):19–22. 29271086 10.1111/tct.12743

[26] Giroux M, Pélissier-Simard L. Shedding light on autistic traits in struggling learners: A blind spot in medical education. Perspectives on Medical Education (Perspect Med Educ). 2021;10(3):180–6. 33611772 10.1007/s40037-021-00654-z PMC8187540

[27] Shaw SCK, Grosjean B, McCowan S, Kinnear M, Doherty M. Autistic role modelling in medical education. Educ Prim Care. 2022;33(2):128–9. 34859733 10.1080/14739879.2021.1996277

[28] Shaw SCK, Doherty M, Anderson JL. The experiences of autistic medical students: A phenomenological study. Medical Education (Med Educ). 2023;57:971–9. 37264701 10.1111/medu.15119

[29] Doherty M, McCowan S, Shaw SC. Autistic SPACE: a novel framework for meeting the needs of autistic people in healthcare settings. Br J Hosp Med. 2023;84(4):1–9. 10.12968/hmed.2023.0006 37127416

[30] Foucault M, Sheridan AM. The Birth of the Clinic: An archaeology of medical perception Vol. 10. 3rd ed. Milton: Routledge; 1963.

[31] Happé F, Frith U. Annual Research Review: Looking back to look forward – changes in the concept of autism and implications for future research. Child Psychology Psychiatry. 2020;61(3):218–32. 10.1111/jcpp.13176 31994188

[32] Meeks LM, Jain NR. Accessibility, inclusion, and action in medical education: lived experiences of learners and physicians with disabilities. Washington, DC: Association of American Medical Colleges; 2018.

[33] Shaw A. Inclusion of disabled higher education students: why are we not there yet? Int J Incl Educ. 2021;28(6):820–38. 10.1080/13603116.2021.1968514.

[34] Woolf K, Potts HWW, McManus IC. Ethnicity and academic performance in UK medical education: a systematic review and meta-analysis. BMJ. 2011;342:d901. 21385802 10.1136/bmj.d901 PMC3050989

[35] VanMatre RM, Nampiaparampil DE, Curry RH, Kirschner KL. Technical standards for medical school admission: the need for a national standard. Acad Med. 2004;79(4):305–9.

[36] Shakespeare T. The social model of disability. In: Davis LJ, editor. The disability studies reader. 5th ed. New York: Routledge; 2017. p. 195–203.

[37] Burgstahler S. Universal design in higher education: promising practices. Cambridge, MA: Harvard Education Press; 2015.

[38] Oliver M. Understanding disability: from theory to practice. London: Macmillan International Higher Education; 1996.

[39] Dolmage J. Academic ableism: disability and higher education. Ann Arbor, MI: University of Michigan Press; 2017.

