# Supplementary material for: Navigating medical school with autism: a systematic review exploring student experiences & support provision in the United Kingdom
Source: BMC Med Educ. 2025 Jul 1;25:927. doi: 10.1186/s12909-025-06866-9 (PMC12210550; doi:10.1186/s12909-025-06866-9)
Supplement: Supplementary file 1 — Supplementary Material 1. [file 12909_2025_6866_MOESM1_ESM.docx]

**Appendices**

Appendix 1: full search strategies conducted on the 19th of May

***Scopus***

Scopus was searched on the 19th of May using the following search criteria:

( TITLE-ABS-KEY ( autis* OR asd OR asperger* OR neurodiver* ) ) AND ( TITLE-ABS-KEY ( "medical education" OR medicine OR "medical school" OR "junior doctors" OR "medical students" OR "undergraduate medical education" OR "clinical practice" OR "neurodiversity medical education" OR physicians ) ) AND ( TITLE-ABS-KEY ( "staff faculty development training" OR "continuing professional development" OR cpd OR faculty OR student AND support OR university OR "clinical competence" ) ) AND PUBYEAR > 2009 AND PUBYEAR < 2025

This search strategy was adapted from the generic search strategy stated above. They were each inputted as separate lines within scopus, each line saved in the ‘saved searches’ folder and then combined using AND.

For example,

Each word of the first line was inputted into the advanced search option of Scopus as a separate line. e.g.:

autis* OR

asd OR

asperger* OR

neurodiver*

On the drop-down options on the left hand side of each line we specified the field of ‘title, abstract, and keyword’ for each word. The search was then saved to our Scopus account as a search and labelled.

This process was then repeated with the next two lines of the strategy.

In the saved searches section of Scopus, we then selected the 3 lines and clicked ‘combine with AND’. With the results of this search, we then used the ‘refine search’ function on the left-hand side of Scopus to apply a filter of 2009-2025. This search yielded 164 papers. This search was then saved as a full search and labelled as such.

The results were exported to Rayyan as an RIS file.

***APA PsychINFO (Via EBSCOHost)***

PsychINFO was searched on the 19th of May 2024.

The search strategy was adapted from the generic search strategy stated above.

Using the advanced search feature in PsychINFO, each line of the search strategy was inputted individually to account for the different filters necessary.

To search for the title, the filter is TI.

To search the abstract, the filter is AB

To search for key descriptors, the filter is DE.

Each word of the first line was inputted into the advanced search option of PsychINFO as a separate line with one filter applied. e.g.:

autis* OR TI

asd OR TI

asperger* OR TI

neurodiver* TI

This process was repeated using the filters AB and DE.

As each filter had to be applied separately, the search for line 1 looked like this:

TI (autis* OR ASD OR Asperger* OR neurodiver*) OR AB (autis* OR ASD OR Asperger* OR neurodiver*) OR DE autism OR DE autistic OR DE asd OR DE autism spectrum disorders OR DE asperger’s OR DE asperger’s syndrome OR DE autistic disorder DE asperger syndrome OR DE neurodivergent OR DE neurodiverse OR DE neurodiversity OR DE neurodivergence OR DE asd intervention

This process was repeated with the second and third lines of our search strategy:

TI (medical education OR medicine OR medical school OR junior doctors OR medical students OR “undergraduate medical education” OR “clinical practice” OR “neurodiversity medical education”) OR AB (medical education OR medicine OR medical school OR junior doctors OR medical students OR “undergraduate medical education” OR “clinical practice” OR “neurodiversity medical education”) OR DE medical OR DE healthcare

TI (“staff faculty development training” OR “continuing professional development” OR CPD OR faculty OR “student support” OR university) OR AB (“staff faculty development training” OR “continuing professional development” OR CPD OR faculty OR “student support” OR university) OR DE career development OR DE higher education

Each line was saved individually in the ‘saved searches’ folder and then combined using AND.

With the results of this search, we then used the ‘limiters’ function on the left hand side of PsychINFO to apply a filter of 2009-2024. This search yielded 71 papers. This search was then saved as a full search and labeled as such.

The results were exported to Rayyan as an RIS file.

***Web of Science***

Web of Science (core collection) was searched on the 19th of May using the following search criteria:

TS=(autis* OR ASD OR Asperger* OR neurodiver*) AND TS=(medical education OR medicine OR medical school OR junior doctors OR medical students OR “undergraduate medical education” OR “clinical practice” OR “neurodiversity medical education” OR physicians) AND TS=(“staff faculty development training” OR “continuing professional development” OR CPD OR faculty OR “student support” OR university OR “clinical competence”) and English (Languages) and 2024 or 2023 or 2022 or 2021 or 2020 or 2019 or 2017 or 2018 or 2016 or 2015 or 2014 or 2013 or 2012 or 2011 or 2010 (Publication Years)

This search strategy was adapted from the generic search strategy stated above. They were each inputted as separate lines within scopus, each line saved in the ‘saved searches’ folder and then combined using AND.

For example,

Each word of the first line was inputted into the advanced search option of Web of Science as a separate line. e.g.:

autis* OR

asd OR

asperger* OR

neurodiver*

On the drop-down options on the left-hand side of each line we specified the field of Topic (TS) for each word. The search was then saved to our Web of Science account as a search and labeled.

This process was then repeated with the next two lines of the strategy.

In the saved searches section of Web of Science, we then selected the 3 lines and clicked ‘combine with AND’. With the results of this search, we then used the ‘refine results’ function on the left-hand side of Web of Science to apply a filter of 2010-2024. This search yielded 181 papers. This search was then saved as a full search and labelled as such.

The results were exported to Rayyan as an RIS file.

***ERIC (via Proquest)***

ERIC (via Proquest) was searched on the 19th of May using the following search criteria:

((title(autis* OR ASD OR asperger* OR neurodiver*) OR abstract(autis* OR ASD OR asperger* OR neurodiver*) OR if(autis* OR ASD OR asperger* OR neurodiver*) OR MAINSUBJECT.EXACT("autism") OR MAINSUBJECT.EXACT("autistic psychopathy") OR MAINSUBJECT.EXACT("autism spectrum disorders") OR MAINSUBJECT.EXACT("asperger syndrome") OR MAINSUBJECT.EXACT("neurodivergent") OR MAINSUBJECT.EXACT("neurodiverse") OR MAINSUBJECT.EXACT("neurodivergence")) AND (title("medical education" OR "medicine" OR "medical school" OR "junior doctors" OR "medical students" OR "undergraduate medical education" OR "clinical practice" OR "neurodiversity medical education") OR abstract("medical education" OR "medicine" OR "medical school" OR "junior doctors" OR "medical students" OR "undergraduate medical education" OR "clinical practice" OR "neurodiversity medical education") OR if("medical education" OR "medicine" OR "medical school" OR "junior doctors" OR "medical students" OR "undergraduate medical education" OR "clinical practice" OR "neurodiversity medical education") OR MAINSUBJECT.EXACT("medical school faculty")) AND (title("staff faculty development training" OR "continuing professional development" OR "CPD" OR "faculty" OR "student support" OR "university") OR abstract("staff faculty development training" OR "continuing professional development" OR "CPD" OR "faculty" OR "student support" OR "university") OR if("staff faculty development training" OR "continuing professional development" OR "CPD" OR "faculty" OR "student support" OR "university") OR MAINSUBJECT.EXACT("staff improvement") OR MAINSUBJECT.EXACT("student welfare"))) AND pd(20100101-20240510)

This search strategy was adapted from the generic search strategy stated above. They were each inputted as separate lines within ERIC, each line saved in the ‘saved searches’ folder and then combined using AND.

This search strategy looks a bit different to our others because ERIC has a ‘thesaurus’. We inputted each of the key words from our generic search strategy into this thesaurus to see whether there were any alternative words that would support our search in ERIC. These additional terms were added to the search strategy using the ‘MAINSUBJECT.EXACT’ function.

Using the advanced search feature in ERIC, each line of the search strategy was inputted individually to account for the different filters necessary.

To search for the title, the filter is title.

To search the abstract, the filter is abstract.

To search for identifiers (keywords) , the filter is if.

Each word of the first line was inputted into the advanced search option of ERIC as a separate line with one filter applied. e.g.:

autis* OR title

asd OR title

asperger* OR title

neurodiver* title

The additional words found through the thesaurus were then also searched here as separate lines using the MAINSUBJECT.EXACT filter option.

The search was then saved to our ERIC account as a search and labeled.

This process was then repeated with the next two lines of the strategy.

In the saved searches section of ERIC, we then selected the 3 lines and clicked ‘combine with AND’. With the results of this search, we then used the ‘refine results’ function on the left hand side of ERIC to apply a filter of 2010-2024. This search yielded 7 papers. This search was then saved as a full search and labeled as such.

The results were exported to Rayyan as an RIS file.

***Medline (via Ovid)***

When preparing the search, we ticked the following options on the ‘select resource(s) to search’ Ovid pop up:

- Ovid MEDLINE(R) and Epub Ahead of Print, In-Process, In-Data-Review & Other Non-Indexed Citations, Daily and Versions 1946 to May 10, 2024
- Ovid MEDLINE(R) Epub Ahead of Print and In-Process, In-Data-Review & Other Non-Indexed Citations and Daily May 10, 2024
- Ovid MEDLINE(R) and In-Process, In- Data-Review & Other Non-Indexed Citations 1946 to May
- 10, 2024
- Ovid MEDLINE(R) ALL 1946 to May 10, 2024
- Ovid MEDLINE(R) and Epub Ahead of Print, In-Process, In-Data-Review & Other Non-Indexed Citations and Daily 2020 to May 10, 2024
- Ovid MEDLINE(R) and Epub Ahead of Print, In-Process, In-Data-Review & Other Non-Indexed Citations and Daily 2020 to May 10, 2024
- Ovid MEDLINE(R) 1946 to April Week 5 2024
- Ovid MEDLINE(R) 1996 to April Week 5 2024
- Ovid MEDLINE(R) 1946 to 1995
- Ovid MEDLINE(R) Epub Ahead of Print • May 10, 2024
- Ovid MEDLINE(R) Daily Update May 10, 2024
- Ovid MEDLINE(R) 2020 to April Week 5 2024

Using the ‘multi-field search’ function, Medline (via Ovid) was searched on the 19th of May.

This search strategy was adapted from the generic search strategy stated above.

On the drop-down options on the left hand side of each line we specified the field of abstract (ab.) for each word, each inputted as separate lines within Medline.

For example,

Each word of the first line was inputted into the multi-field search option of Medline as a separate line. e.g.:

autis* OR

Autism Spectrum Disorder

ASD OR

Asperger* OR

neurodiver*

After saving this search to our account as the ‘no limits’ search, we used the ‘limits’ section of Medline to select the following limits:

- Full Text
- Abstracts
- English Language
- 2010- Current

This process was then repeated with the next two lines of the strategy.

So, the saved searches/search history looked like this:

1. (autis* or Autistic Spectrum Disorder or ASD or Asperger* or neurodiver*).ab.
2. limit 1 to (abstracts and english language and-full text and yr="2010 -Current")
3. (medical education or medic* or medical school or doctor* or medical student*).ab.
4. limit 3 to (abstracts and english language and full text and yrs"2010 -Current")
5. (staff faculty development training or continuing professional development or CPD or faculty or student support or university).ab.
6. limit 5 to (abstracts and english language and full text and yr="2010 -Current")

We then selected the 3 lines of the strategy with limits and clicked ‘combine with AND’.

The final search command was:

2 and 4 and 6

This search yielded 27 papers. This search was then saved as a full search and labeled as such.

The results were exported to Rayyan as an RIS file.

***Embase (via Ovid)***

When preparing the search, we ticked the following options on the ‘select resource(s) to search’ Ovid pop up:

- Embase 1996 to 2024 Week 18
- Embase 1988 to 2024 Week 18
- Embase 1980 to 2024 Week 18
- Embase 1974 to 2024 Week 18
- Embase 1974 to 2024 May 09
- Embase 1988 to 1995

Using the ‘multi-field search’ function, Embase (via Ovid) was searched on the 19th of May.

This search strategy was adapted from the generic search strategy stated above.

On the drop-down options on the left hand side of each line we specified the field of abstract (ab.) for each word, each inputted as separate lines within Embase.

For example,

Each word of the first line was inputted into the multi-field search option of Embase as a separate line. e.g.:

autis* OR

Autism Spectrum Disorder

ASD OR

Asperger* OR

neurodiver*

After saving this search to our account as the ‘no limits’ search, we used the ‘limits’ section of Medline to select the following limits:

- Full Text
- Abstracts
- English Language
- 2010- Current

This process was then repeated with the next two lines of the strategy.

So, the saved searches/search history looked like this:

1. (autis* or Autistic Spectrum Disorder or ASD or Asperger* or neurodiver*).ab.
2. limit 1 to (abstracts and english language and-full text and yr="2010 -Current")
3. (medical education or medic* or medical school or doctor* or medical student*).ab.
4. limit 3 to (abstracts and english language and full text and yrs"2010 -Current")
5. (staff faculty development training or continuing professional development or CPD or faculty or student support or university).ab.
6. limit 5 to (abstracts and english language and full text and yr="2010 -Current")

We then selected the 3 lines of the strategy with limits and clicked ‘combine with AND’.

The final search command was:

2 and 4 and 6

This search yielded 58 papers. This search was then saved as a full search and labeled as such.

The results were exported to Rayyan as an RIS file.
